# Natural history of conjugated bilirubin trajectory in neonates following parenteral nutrition cessation

**DOI:** 10.1186/s12887-014-0298-z

**Published:** 2014-12-10

**Authors:** Nisha Mangalat, Cynthia Bell, April Graves, Essam M Imseis

**Affiliations:** Department of Pediatrics, Saint Louis University School of Medicine, 1465 S. Grand Blvd, Saint Louis, MO 63104 USA; Department of Pediatrics, University of Texas Medical School at Houston, 6431 S. Fannin, Suite 500, Houston, TX 77030 USA

**Keywords:** Neonatal cholestasis, Parenteral nutrition

## Abstract

**Background:**

There is little published data regarding the rate of bilirubin clearance in newborns following total parenteral nutrition (TPN) cessation, particularly in the neonatal intensive care unit (NICU) population without intestinal failure.

**Methods:**

The primary aim of this retrospective chart review was to determine the duration and severity of bilirubin elevation in neonates without intestinal failure. Secondary aims were to determine factors that would influence the duration and severity of this biochemical elevation. The authors conducted a retrospective chart review of all infants receiving TPN for ≥ 21 days and with elevated conjugated bilirubin (CB) ≥3 mg/dL upon TPN cessation in a tertiary care NICU from January 1, 2008 to December 1, 2010. Patients with known causes of liver disease or without laboratory values at least four weeks after PN cessation were excluded. Time to maximum conjugated bilirubin (maxCB) post TPN cessation and normalization were the primary outcomes. Secondary factors including number/timing of sepsis events, ethnicity, and ursodiol use were also evaluated.

**Results:**

Forty three infants met inclusion criteria. The majority of patients had increased CB post TPN cessation (“up” group; 27/43, 63%) with maxCB reached 13 days (SD ± 10.3) after TPN cessation. The majority of the cohort achieved normalization of the bilirubin prior to discharge (28/43, 65%). There was no difference in rate of normalization (p = 0.342) between the “up” group (59%) and the group of patients whose bilirubin trended downward following PN cessation (“down” group, 75%). There were no differences between the two groups with respect to gestational age at birth, birth weight, number of sepsis events, gram negative sepsis events, or intestinal resection. Only 30% of Hispanic patients had increased CB post TPN cessation compared to the majority (71%) of non-Hispanic patients. The maxCB of those that had complete normalization was significantly lower value than the maxCB of those that did not normalize (p = 0.016).

**Conclusions:**

Nearly two-thirds of infants experience a rise in serum bilirubin following PN cessation that can last for weeks, but cholestasis generally improves with time in the majority of infants.

**Electronic supplementary material:**

The online version of this article (doi:10.1186/s12887-014-0298-z) contains supplementary material, which is available to authorized users.

## Background

Liver disease associated with prolonged parenteral nutrition (PN) is a well-recognized phenomenon. Evidence of biochemical liver injury may be present as early as 2 weeks after initiation of parenteral nutrition [1]. Numerous studies have shown that patients with parenteral nutrition associated liver disease (PNALD) have significant morbidity and mortality [2]. Moreover, in neonates with short bowel syndrome, reduction of PNALD is associated with improvement in survival and outcomes [3]. The mechanism of PN-associated liver disease is not entirely known and is likely multi-factorial. Potential causes include loss of epithelial barrier function leading to passage of enteric organisms into the hepatic circulation with subsequent endotoxin and inflammatory cytokine release [4]. Additionally, alteration in expression of proteins involved with canalicular bile acid transport, such as multi-drug resistance transporters (MDR1 and MDR2), may contribute to the liver dysfunction seen in individuals receiving PN. The use of intravenous lipid emulsions has also been shown to place patients at higher risk for liver disease and is an independent risk factor for the development of PNALD [5,6]. Studies have indicated that strategies such as lipid minimization may be useful in preventing PNALD [7,8].

While it is clear that PN has significant effects on neonatal morbidity and mortality, the progression of liver disease after parenteral nutrition has been discontinued has not been well studied. The general assumption is that PN cholestasis will improve once full enteral nutrition has been achieved. However, one study by Yang et al. reported that in pediatric patients with short bowel syndrome ALT and bilirubin worsened for several weeks after cessation of PN and normalized 8 weeks after PN discontinuation [9].

This study will attempt to describe the natural history of PNALD in infants without intestinal failure following cessation of PN. Further, we aim to identify host and nutritional factors associated with resolution of PNALD.

## Methods

### Study population

All patients admitted to the Neonatal Intensive Care Unit (NICU) at Children’s Memorial Hermann Hospital receiving parenteral nutrition (PN) for greater than or equal to 21 days from January 1, 2008 to December 31, 2010 were screened for eligibility. For purposes of this study, patients were included if they had a diagnosis of cholestasis with a bilirubin ≥ 3 mg/dL during their hospitalization in our neonatal intensive care unit (NICU). Those infants who remained on PN prior to discharge with insufficient laboratory follow up (sufficient laboratory follow-up defined as having laboratory data available for at least four weeks post PN cessation), those with other identified etiologies for liver disease, and those with cyanotic congenital heart disease were all excluded from this study. Institutional Review Board approval was obtained from the University of Texas Health Science at Houston and Children’s Memorial Hermann Hospital prior to the initiation of this retrospective chart review.

### Data collection

Demographic data (including date of birth, gender, gestational age, ethnicity, birth weight, presence or absence of intrauterine growth retardation); gastrointestinal related medical diagnoses (number of episodes of necrotizing enterocolitis, intestinal resection); nutritional/growth parameters (days receiving PN, days receiving parenteral lipid >2 g/kg/day, date of initiation of enteral feeds, type of enteral nutrition, route of feeding administration), cholestasis history (date and value of maximal conjugated bilirubin (CB), date of resolution of cholestasis defined as CB < 2), and infectious history (number of episodes of sepsis/suspicion of sepsis, type of sepsis event, organism) were collected.

### Statistical analysis

Patients were classified into two groups based on whether CB increased (“up” group) or decreased (“down” group) immediately after PN cessation. Primary outcome was the percentage of patients who reached normalized CB < 2 mg/dL within 4 weeks of PN cessation in each group, tested by Fisher’s exact test. Nutritional and host factors were compared between groups using Wilcoxon rank-sum test for continuous variables and Chi-squared or Fisher’s exact test for categorical variables. Time to CB normalization was estimated by Kaplan-Meier method and tested by log-rank test.

## Results

### Patients

A total of 341 charts of NICU patients receiving PN for ≥21 days were screened for this study. Based on the criterion of having CB ≥ 3 mg/dL during hospitalization, 164 (48%) patients were eligible. Of these, 43 met all inclusion criteria with no evidence for any other identified cause of liver disease and with sufficient laboratory monitoring for analysis. The vast majority were excluded due to insufficient bilirubin values available for review four weeks after PN cessation. The mean gestational age was 27.3 weeks +/− 3.9 SD (median 26 weeks, min 23–max 38). Patients were observed for a median of 120 days (min 63–max 311) after PN cessation.

### Overall trends of CB after PN cessation

**Figure 1:**
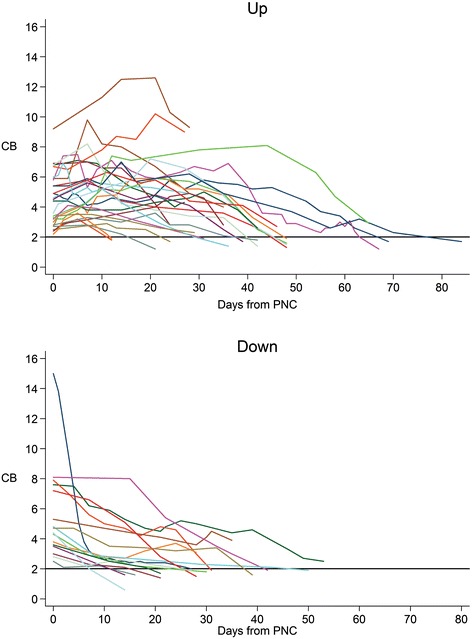
**Bilirubin trajectory of up and down groups.**

Two distinct trends in bilirubin trajectories were noted in our series of patients. Of the 43 patients meeting inclusion criteria, 27 of the patients (63%) had an increase in CB after PN cessation and 16 patients (37%) had a decrease in CB without any further rise in CB upon discontinuation of PN (Figure 1). Overall, 28 patients in the study group (65%) eventually achieved normalization of the CB during the available follow up period (at least four weeks of laboratory data). Among patients in the “up” group, 16 (59%) had complete normalization of the CB, while the remainder had an ongoing downward trend of CB but had not yet reached defined normal values within the observation period. In the “down” group, 12 (75%) eventually had complete normalization of the bilirubin within the study period, while the remainder also had downward trending CB. There was no significant difference in the rate of normalization between the two groups (p = 0.342). The baseline mean value of CB at time of PN cessation was 4.5 ± SD 1.8 mg/dL in the “up” group which was comparable to 5.5 ± SD 3.1 mg/dL in the “down” group. While maximal CB was also similar in the two groups (6.2 ± SD 2.3 mg/dL in “up” group vs. 5.5 ± SD 3.1 mg/dL in “down” group), the rate of CB decline was slower in the “up” group due to longer time to minimum CB (39.1 ± SD 17.3 days) compared to the down group (28.4 ± SD12.3 days). Similarly, by Kaplan-Meier estimates, median time to normalization was significantly longer in the “up” group (48 days, CI: 39–48) compared to “down” group (30 days, CI: 21–42, p = .025 in the down group, despite similar rates of bilirubin decline.

Demographic data between the “up” and “down” groups were similar, though there were fewer Hispanic infants in the “up” group compared to the “down” group (33% vs. 66%). There were no differences in gestational age, birth weight, gender or prevalence of intrauterine grown retardation (IUGR) between groups (Table 1).

**Table 1:** **Group comparisons**

	**Up group, n = 27**	**Down group, n = 16**	
Gender (M)	20 (74%)	10 (63%)	p = 0.638
Gestational age	27.26 ± 3.59	27.25 ± 4.60	p = 0.637
Birth weight, g	992 ± 613	984 ± 663	p = 0.315
Ethnicity (n)			
Caucasian	10	5	p = 0.185
African American	11	3	
Hispanic	3	6	
Asian	1	0	
Unknown	2	2	
Length of stay, days	129.52 ± 67.67	141.31 ± 58.11	p = 0.499
Duration of PN, days	53.96	49.63	p = 0.421
Intrauterine growth retardation (IUGR)	2	1	p = 1.00
Age at EN initiation, days	11.15 ± 13.80	24.13 ± 22.53	p = 0.037
Type of EN (n)			p = 0.573
BM	8	3	
Standard formula	13	11	
Protein hydrosylate	2	0	
Amino Acid	4	2	
Route of feeding administration (n)			p = 0.196
PO/bolus	25	13	
Continuous	1	0	
Combination	1	3	
Ursodiol use (n)	19	8	p = 0.182
Start & stop before PN cessation			
Start before, stop after PN cessation	9	2	
Start and stop after PN cessation	13	4	
Sepsis (n)	14	8	p = 0.578
Occurred before PN cessation	13	5	p = 0.117
Occurred after PN cessation	1	2	
Occurred before and after PN cessation	0	1	
Gram negative sepsis	5	3	p = 0.642
Occurred before PN cessation	5	2	p = 0.375
Occurred after PN cessation	0	1	
Rate of normalization (CB < 2 mg/dL)	16	12	p = 0.295

### Intestinal resection

There were no differences in the rate of any type of intestinal resection between groups. There were three infants with intestinal resection were noted in the “up” group and two infants with intestinal resection in the “down” group. All five of these infants had small bowel resections, and two of these had additional colonic resection, one in the “up” and the other in the “down” group.

### Nutrition

There were no differences in types of enteral nutrition, and modalities of enteral feedings (bolus feeds, continuous feeds, or combination of both) between the two groups. There was significant difference in the age of EN initiation, with younger age of EN initiation noted in the up group (11.5 days) versus down group (24.13 days, p = 0.037). There were no differences in composition of parenteral nutrition between the two groups (Table 1). A typical parenteral nutrition prescription for the study time period is attached as an Additional file 1.

### Sepsis

There were no differences in number of episodes of sepsis or timing of sepsis in relation to discontinuation of PN in the two groups (p = 0.117, Table 1). There were also no differences noted when gram negative sepsis events were analyzed separately for the two groups. There were no differences in the number of episodes of necrotizing enterocolitis between the groups (p = 1.0).

### Ursodiol use

**Figure 2:**
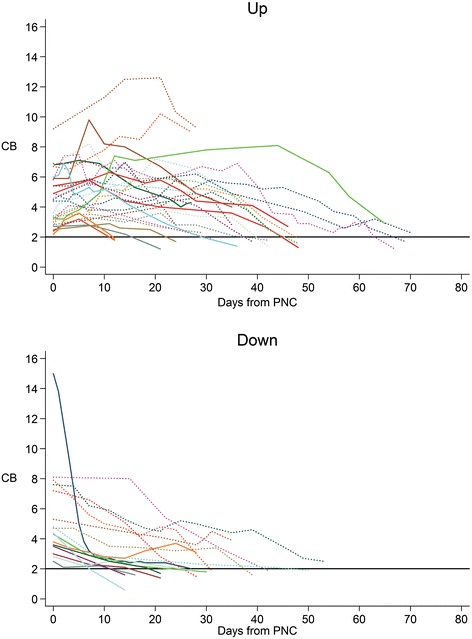
**No difference in decline of CB based on Ursodiol use.**

Of patients in the “up” group, 19/27 (70%) were taking ursodiol compared with 8/16 (50%) in the “down” group; this difference was not statistically significant. There was no difference in the initiation and discontinuation of ursodiol treatment between the two groups (Figure 2).

### Normalization group

Twenty eight of the 43 total patients (65%) with evidence of PN-associated cholestasis at time of PN cessation eventually had complete normalization of the CB during the study period. Among those that eventually normalized compared to those that did not have normalization, there was significantly lower gestational age (25.6 weeks versus 30.2 weeks, p <0.001) and birth weight (730 g versus 1.47 kg, p < 0.001) in the normalization group. Baseline CB and maximum CB were lower in the group that normalized. The maximum CB that eventually normalized during the course of this study was 8.2 mg/dL.

## Discussion

Neonates requiring PN are at risk of significant cholestatic liver disease. While this injury can occur early and often in young infants, it is generally felt to be reversible in most infants following discontinuation of PN. While we noted a high percentage of cholestatic liver disease in our patient population, the incidence of cholestasis in our study is consistent with those reported by others [10]. Since our study period, the use of parenteral lipid minimization and ethanol lock therapy in select infants has resulted in significant reduction in our institution’s rate of PN-associated cholestasis. Nevertheless, when cholestasis develops, it appears to resolve in most despite large differences in bilirubin trajectories in infants following this PN cessation. Our series confirmed a phenomenon that is often noted clinically—that rise in bilirubin may initially occur in a significant percentage, if not the majority, of infants following PN cessation. This rise may warrant further workup in the appropriate clinical setting, such as the need to exclude diagnoses such as gallstones or biliary atresia, if the child’s age and clinical picture cannot confidently rule these conditions out. However, knowledge of the natural history upon cessation of PN can reduce the need for invasive, costly, and often unnecessary testing, and provide reassurance to caregivers and parents that these other investigations may be unnecessary in most children.

We analyzed a number of factors that may have effects on bilirubin metabolism and clearance following PN cessation, but few differences were noted comparing the “up” and “down” groups. Normalization of bilirubin values was more often seen in infants who were more premature, but this may be due to longer hospitalization and closer follow up in a higher risk group. In our study, use of ursodiol was more likely to be associated with an increase in bilirubin following PN cessation rather than a decrease, although our results were not statistically significant. Ursodiol is often used in children with cholestatic liver disease since its choleretic effects may enhance bilirubin clearance, although there is little evidence supporting its use. Hispanic infants were not more likely to have a rise in bilirubin after PN cessation than non-Hispanic patients. This was surprising since children of Hispanic descent are at increased risk for other hepatobiliary disease including steatohepatitis and gallbladder disease. Interestingly, chronic adult cholestatic conditions, including primary biliary cirrhosis and primary sclerosing cholangitis, may be less common in individuals of Hispanic descent versus other major ethnic groups. We investigated Hispanic race as a potential confounder but no other factors recorded in the study showed associated with Hispanic race. Nevertheless, it is possible that this observation was random false positive due to Type 1 error inherent in all statistical analyses.

Our study did have some limitations. Given the observational nature, infant care was not standardized and sample size was not large enough to determine effects of variables such as demographics or specific treatments of this biochemical observation in bilirubin following PN cessation. Furthermore, infants were not followed longitudinally to determine if other manifestations of liver disease such as poor weight gain and growth were more prevalent in those infants with prolonged cholestasis or worsening cholestasis following PN cessation. Therefore, results of this study are only applicable to infants with a similar hospital course and should be noted by physicians caring for these patients.

## Conclusions

While our findings appear to confirm that there are two distinct bilirubin curves after PN cessation, the eventual biochemical resolution of cholestasis appears to occur in majority of infants four weeks post PN cessation. It is important to note that normalization of bilirubin values does not always reflect normalization of liver histology. In fact, a number of publications have demonstrated persistence of liver fibrosis despite resolution or improvement in cholestasis following PN cessation [11-13]. Nevertheless, further investigation of these individuals is unlikely to be of significant help in the majority. Longitudinal observation in most infants following PN cessation is sufficient, inasmuch as a rise in serum bilirubin can now be viewed as a common occurrence before resolution of cholestasis.

## Additional file

Additional file 1:
**Example of PN prescription used in the NICU during study period.** An example of typical PN prescription used during the study period is included. On day of life 1, infants were typically started at 1 gm/kg/day of intralipid (IL) and increased daily to 3 gm/kg/day if the triglycerides were less than 200–250 mg/dL. Most infants tolerated escalation in IL to 3 gm/kg/day by day 4 of life. Since our study period, the use of parenteral lipid minimization and ethanol lock therapy in select infants has resulted in significant reduction in our institution’s rate of PN-associated cholestasis.
