# Supplementary material for: Natural history of conjugated bilirubin trajectory in neonates following parenteral nutrition cessation
Source: BMC Pediatr. 2014 Dec 10;14:298. doi: 10.1186/s12887-014-0298-z (PMC4275980; doi:10.1186/s12887-014-0298-z)
Supplement: Additional file 1: — Example of PN prescription used in the NICU during study period. An example of typical PN prescription used during the study period is included. On day of life 1, infants were typically started at 1 gm/kg/day of intralipid (IL) and increased daily to 3 gm/kg/day if the triglycerides were less than 200–250 mg/dL. Most infants tolerated escalation in IL to 3 gm/kg/day by day 4 of life. Since our study period, the use of parenteral lipid minimization and ethanol lock therapy in select infants has resulted in significant reduction in our institution’s rate of PN-associated cholestasis. [file 12887_2014_298_MOESM1_ESM.docx]

# *TPN/IL ORDERS MUST BE RE-WRITTEN EVERY MONDAY & THURSDAY* WEIGHT: ___________ KG

_______ ml/kg/day 20% Lipid Emulsion x _______ kg/24 hrs = Rate of IL Infusion ________ ml/hr ( ______ g/kg/day)

_______ ml/kg/day Parenteral Nutrition x _______ kg/24 hrs = Rate of TPN Infusion ________ ml/hr

🞏 **STARTER TPN**

**(< 1000g BW)**

per 100 ml

## 🞏 PREMATURE

per 100 ml

## 🞏 TERM

per 100 ml

## 🞏 NON-STANDARD

per 100 ml

**DAILY GOALS**

_____ g protein/kg

_____ GIR mg/kg/min

_____ mEq Na/kg

_____ mEq K/kg

_____ mMol PO_4_/kg

_____ mEq Ca/kg

_____ mEq Mg/kg

Amino acids ______ g

Dextrose ______ %

Na ______ mEq

K ______ mEq

PO_4_ ______mMol

Ca ______ mEq

Mg ______ mEq

Anions: (choose one)

🞏 Maximum Chloride

🞏 Maximum Acetate

🞏 _______:_______

Chloride : Acetate

Amino acids 2.2 g

Dextrose 12.5 %

Na 2.6 mEq

K 2 mEq

PO_4_ 1 mMol

Ca 2.5 mEq

Mg 0.5 mEq

Anion ratio:

1:1 chloride: acetate

Amino acids 2.8 g

Dextrose 12.5 %

Na 2.6 mEq

K 2 mEq

PO_4_ 1.3 mMol

Ca 3.1 mEq

Mg 0.5 mEq

Anion ratio:

1:1 chloride: acetate

Amino Acids 2 g

Dextrose ______%

No Electrolytes

No Cysteine

No MVI

No Trace Elements

# CHANGES:

🞏 **Trace Elements for Cholestasis (DB>4)**

NT4 + SE on Mon & Thurs Only

Remaining days give only:

Zinc 400 micrograms/kg

Chromium 0.2 micrograms/kg

Selenium 2 micrograms/kg

🞏 **Trace Elements for Renal Dysfunction**

NT4 + SE on Mon & Thurs Only

Remaining days give only:

Zinc 400 micrograms/kg

Copper 20 micrograms/kg

Manganese 1 microgram/kg

**ADDITIONS:**

🞏 **Trace Elements for exclusive PN >30 days**

Molybdenum 0.25 micrograms/kg

Carnitine 10 mg/kg

**Additional Zinc for GI losses**

🞏 400 mcg/kg/day infants <2.5 kg

🞏 250 mcg/kg/day infants >2.5 kg

🞏 100 mcg/kg/day infants >3 mo CGA

# MUST CHECK ONE

🞏 Heparin 0.25 units/ml (only with central line)

🞏 No Heparin (with peripheral line)

🞏 Heparin ______ units/ml

**Other additives**

___________________________

___________________________

ADDED PER PROTOCOL

Multivitamins (wt based) 🞏 Do not add

Trace Elements NT-4 + Selenium (wt based) 🞏 Do not add

L-Cysteine 20 mg/g amino acids 🞏 Do not add

# CALCULATIONS

Value per kg/day = Value

ml/kg/day PN 100 ml

GIR: (__%dextrose)(___ml/kg/day) ÷ 144 = ___mg/kg/min

#### Calorie Calculations :

_____ ml/kg PN x ____g AA x 4 kcal/100 = _______

_____ ml/kg PN x ___%Dex x 3.4 kcal /100 = _______

_____ ml/kg IL x 2 kcal/ml = _______

Total kcal/kg _______

### FLUID CALCULATIONS

_______ml/kg/day PN

_______ml/kg/day IL

_______ml/kg/day IVF

_______ml/kg/day IV Med. Drips

_______ml/kg/day enteral feeds

_______TOTAL FLUIDS

Signature Printed Name Phone/pager Provider # Date/Time


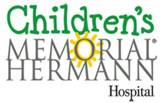


1. All orders must be received in pharmacy by 3pm.
2. The PN bag will contain 75ml of overfill.

# Neonatal Parenteral Nutrition Orders

# Parenteral Nutrition Recommendations Back Neonatal

**A. Nutrient Requirements for Term and Preterm Neonates:** 90-115 kcal/kg/day, 2.7–4 g protein/kg/day

### B. ORDERING TPN

1. TPN orders are due by 3:00pm.
2. TPN/IL orders must be **re-written every Monday and Thursday**, & when changes in the concentrations are needed.
3. Order starter TPN upon admission to the NICU for infants <1000g BW.
4. Infants on starter TPN should be changed to non-starter TPN on DOL 1-3.
5. In infants >1000g birth weight, begin TPN on DOL 1 or when the need for TPN is established.
6. Initiate IL (Intralipid 20%) on DOL 1 at 1 g/kg/day (5 mL/kg/day) and advance to goal of 3 g/kg/day as tolerated.
7. Check TG level after IL initiated and after rate increases.

| Dextrose | 3.4 kcal/g |
| --- | --- |
| Amino Acids | 4 kcal/g |
| Fat (20% IL) | 2 kcal/ml  (1 g fat = 5 mL) |

1. Administer TPN & IL by continuous infusion over 24 hours.

#### C. Carbohydrate Administration

1. Initiate about 5-6 mg dextrose/kg/minute.
2. Advance by 2.5% or 1-2 mg/kg/minute up to a maximum of 12 mg/kg/minute.
3. Maximum dextrose concentration in a peripheral line is 12.5%.

#### D. Protein: Amino Acids (Trophamine / Premasol is used for all neonates & infants)

1. Initiate 1-2 g/kg/day. Advance by 1 g/kg/day as tolerated to 3-3.5 g/kg/day.
2. Protein should not exceed 15% of total parenteral nutrition calories.

**E. Electrolytes and Minerals** (after the first few days of life, in stable growing babies)

Sodium 3-4 mEq/kg/day Calcium 2-4 mEq/kg/day

Potassium 2-3 mEq/kg/day Phosphorus 1-2 mMol/kg/day

Chloride 2-4 mEq/kg/day Magnesium 0.5-1 mEq/kg/day

1. Give standard Ca & PO_4_ in most cases [1.6:1 weight (mg) ratio]
2. For mild hypercalcemia (11-12.5 mg/dL, iCa 1.3-1.5 mmol/L) or mild hyperphosphatemia (>9 mg/dL): In general, decrease the amount of the elevated mineral by 20-25% and then recheck the following day. Do not remove Ca/PO_4_ from TPN for mild hypercalcemia/hyperphosphatemia.
3. It is rarely appropriate to remove PO_4_ from TPN for >48 hours without adjusting Ca & monitoring iCa.
4. Standard amounts of PO_4_ can be delivered with as little as 2 mEq/100ml of either Na or K (without using acetate or chloride). If TPN is written with less Na and K such that standard PO_4_ can not be given, Ca should not be given either, except as treatment for hypocalcemia.
5. If the Ca (mEq/100ml) + PO_4_ (mmol/100ml) is < 5, precipitation will usually not be a problem.

**F. Vitamins**: Standard daily dose for weight of MVI-Pediatric. ≤ 2.5 kg: 2 ml/kg/day

> 2.5 kg: 5 ml/day (not weight based)

MVI-Pediatric (per 5 ml):

| A (IU) | D (IU) | E (IU) | C (mg) | Thiamine (mg) | Riboflavin (mg) | Niacin (mg) |
| --- | --- | --- | --- | --- | --- | --- |
| 2300 | 400 | 7 | 80 | 1.2 | 1.4 | 17 |
| B6 (mg) | B12 (mcg) | K (mcg) | Biotin (mcg) | Folacin (mcg) | Pantothenate (mg) |  |
| 1 | 1 | 200 | 20 | 140 | 5 |  |

**G. Trace ELEMENTS**: Standard daily dose for weight of Neotrace-4 + Selenium 2 micrograms/kg/day

| *mcg/kg/day* | Zn | Cu | Cr | Mn | Se |
| --- | --- | --- | --- | --- | --- |
| Infants < 2.5 kg | 375 | 25 | 0.2 | 6.25 | 2 |
| Infants > 2.5 & < 5 kg | 300 | 20 | 0.17 | 5 | 2 |
| Infants > 5 kg | 150 | 10 | 0.1 | 2.5 | 2 |

< 2.5 kg: 0.25 ml/kg/day

> 2.5 and <5 kg: 0.2 ml/kg/day

> 5 kg: 0.1 ml/kg/day

### H. CONVERSIONS & CALCULATIONS

1. GIR: (g glucose/kg/day x 1000) / 1440 = mg/kg/min **or** % glucose x ml/kg/day /144 = mg/kg/min
2. Conversion factors (elemental):

Ca 20mg = 1mEq = 0.5mMol Na 23mg = 1mEq = 1mMol

PO_4_ 31mg = 1mMol K 39mg = 1mEq = 1mMol

Mg 12mg = 1mEq = 0.5mMol Cl 35mg = 1mEq = 1mMol

3. Calculation of phosphate salts: 1 mEq KPO_4_ ≅ 1.5 mEq K and 1mMol PO_4_

1 mEq NaPO_4_ ≅ 1.33 mEq Na and 1 mMol PO_4_

*4/28/2008*
